# Supplementary material for: Virulence beneath the fleece; a tale of foot-and-mouth disease virus pathogenesis in sheep
Source: PLoS One. 2019 Dec 31;14(12):e0227061. doi: 10.1371/journal.pone.0227061 (PMC6938329; doi:10.1371/journal.pone.0227061)
Supplement: Attachment — Submitted filename: Response to reviewers_08Dec19.docx [file pone.0227061.s002.docx]

I believe the authors have done an excellent job in addressing the concerns of the reviewers. However, two minor issues remain. First of all, I think that the addition of the same data in Table format for Figs. 1 and 4 is redundant. The authors should delete the Supplementary tables. Secondly, I do think that the addition of the animal numbers in the figures makes it easier to follow the data and I strongly urge the authors to insert the animal numbers and magnification in Fig. 2, 3 and 5.

**Response:** The supporting information files have been discarded. Animal IDs and magnification have been added to the image panels as suggested.
